# Investigating food insecurity, health lifestyles, and self-rated health of older Canadians living alone

**DOI:** 10.1186/s12889-022-14467-0

**Published:** 2022-12-05

**Authors:** Xiangnan Chai, Junyi Mei

**Affiliations:** Sociology Department, School of Social and Behavioral Sciences, Nanjing University, He’ren Building 359, Xianlin Street, Qixia District, Nanjing, Jiangsu Province People’s Republic of China

**Keywords:** Older Canadians, Living alone, Food insecurity, Physical activity, Cigarette smoking, Alcohol drinking, Self-rated health, Canadian community health survey

## Abstract

**Background and objectives:**

There is a large number of older Canadians living alone, calling for academic attention to the health outcomes of this demographic fact. Although many studies have investigated the association between living arrangements and health, little is known about potential underlying mechanisms regarding how living alone may predict older Canadians’ health. In this study, we address this research gap intending to contribute to offering policy suggestions for older Canadians who live alone.

**Research design and methods:**

We applied Cockerham’s health lifestyle theory to explore to what degree living alone predicts worse health lifestyles and, further, to what degree these lifestyles can explain the association between living alone and older Canadians’ health. We used the 2017–2018 Canadian Community Social Survey (Annual Component) which has a response rate of 58.8%. We focused on respondents aged 60 and above, and the analytical sample size is 39,636.

**Results:**

Older Canadians living alone are more likely to have food insecurity problems and higher possibilities of smoking cigarettes compared to those living with spouses/partners with or without children. Compared to those living with spouses/partners only, the odds of solo-living older Canadians drinking regularly is significantly lower. There also exists a significant difference between older Canadians living alone and their counterparts living with spouses/partners that the former reported lower self-rated health compared to the latter. Moreover, food insecurity and the three health lifestyle variables are significantly associated with respondents’ self-rated health; food insecurity, cigarette smoking, and alcohol drinking can partially explain the difference in self-rated health due to living arrangements.

**Discussion and implications:**

According to our findings, health officials are recommended to pay more attention to food insecurity and heavy smoking problems facing older Canadians who live by themselves. Local communities and other stakeholders are suggested to provide older adults living alone with more opportunities for social engagement and involvement since regular drinking may have played such a role in enhancing social life quality of the aged.

**Supplementary Information:**

The online version contains supplementary material available at 10.1186/s12889-022-14467-0.

## Background

Living alone is one of the most prevalent living arrangements among older adults in many developed societies. In Canada, where population aging is an increasing concern, more than one-quarter of Canadians aged 65 years and above lived by themselves [1]. Other more developed societies, such as Northern and Western Europe [2], the U.K. [3], Australia [4], New Zealand [5], Japan [6], and South Korea [7], have similar proportions of older adults who live alone. Reasons such as gender differences in life expectancy, consistent improvement in older cohorts’ educational attainment, increased social welfare benefits for older adults, and the prevalence of individualistic cultures encouraging independence have contributed to the prevailing trend of solo living in these societies [3, 6, 8–11].

Results from previous studies highlight competing health implications for older adults living alone compared to other living arrangements, most notably living with families [12]. Specifically, living alone may show neutral, positive, or negative effects to health. Some research found no associations between living alone and self-rated physical health [13], depressive symptoms and cognitive impairment [14], and social health [10]. Other studies revealed positive implications of living alone, for example, a less steep descent in mental health among older adults [15] and better current self-rated health [16]. However, a rising number of other studies have found that living alone may result in worse health outcomes, including higher risks of harmful health behaviors such as smoking, unhealthy eating, and less or no exercising [3, 17], worse physical health [3, 18], worse mental health [17, 19–21], and worse emotional health [22]. Possible mechanisms linking living alone to some of these adverse health outcomes include the social exclusion or social disconnection solo-living older adults may face [23–25], and lack of financial assistance [19].

Two specific Canadian contexts warrant the importance of the current research. First, Canada has been experiencing population aging over the past four decades, as indicated by the increased proportion of older adults in its total population [26]. In 2022, the number of Canadians aged 60 and more had reached about 9.970 million, 25.61% of the entire population of Canada [27]. Second, the single-person household was the leading household type in Canada in 2016 [28]. These contextual shifts entail the investigation of health needs of those solo-living older Canadians, from which researchers can provide policymakers with solid evidence-based recommendations in this vein. However, the extent to which living alone is associated with older Canadians’ health, and potential underlying mechanisms remain unclear. Specifically, although those prior scholarly efforts explore why living alone has adverse health outcomes, few have concentrated on the role of health-related lifestyles. In this research, we applied the health lifestyle theory proposed and developed by Cockerham and colleagues [29–33] to investigate the potential linkage between living arrangements (living alone versus other types of living arrangements) and older Canadians’ health.

### Theoretical framework: Cockerham’s health lifestyle theory

People are confronted with many health issues and risks in contemporary society, so they increasingly take health-related lifestyles into great consideration. Theoretical scholarship of classical sociology should not be ignored to understand “health lifestyle.” As Cockerham and associates pointed out [29], Weber distinguished “life choices” and “life chances.” Life choices refer to an individual’s agency to operationalize lifestyle; however, life choices are constrained by life chances that are shaped by the specific social structure an individual is situated. Based on Weber’s framework, Cockerham developed a theoretical framework on health lifestyles that draws on earlier theorists’ works [29, 30, 33]. Specifically, an individual’s life choices or agency is influenced by their socialization process, which is shaped by the person’s demographic characteristics (age, gender, race, etc.), socioeconomic features (class, living conditions, etc.), and collectivities (kinship, religion, etc.). Meanwhile, life chances are embedded in the external social structure where an individual is located. As Bourdieu proposed in *Distinction* [34], the interplay between life choices and life chances further molds an individual’s dispositions to act. Specific dispositions will shape people’s actions and practices in daily lives, such as healthy eating, doing exercise, smoking, and drinking, which finally cast forms of lifestyles, and lifestyles reproduces dispositions and actions on the contrary. Therefore, as Cockerham defined [33], “health lifestyles are collective patterns of health-related behavior based on choices from options available to people according to their life chances” (p.1036).

Previous empirical studies have applied Cockerham’s theory to explore how health lifestyles influence people’s health outcomes. For example, Christensen and Carpiano [35] examined the possible association between social class and obesity among Danish women. Findings indicate that more cultural, economic, and social capital refers to more participation in exercise, which further contributes to women’s lower Body Mass Index (BMI). However, a woman’s cultural capital may also be positively associated with her BMI because of the mechanism of cooking as an indicator of lifestyle at Bourdieu’s meaning. This study reveals the importance of health lifestyles resulting from an individual’s choices under structured chances due to available capital and resources the person can achieve.

Additionally, Cockerham [32] highlights specific sociocultural contexts of health lifestyles because individuals may be confronted with different health problems and risks across societies. Our research concentrates on whether and to what degree older adults’ health lifestyles are associated with their self-rated health. We have further detected whether their health lifestyles or health-related behaviors are possible mediators that can explain the possible association between living arrangements and self-rated health status to a certain but unknown degree.

### Food insecurity issue facing older Canadians and their health lifestyles: physical exercise, cigarette smoking, and alcohol drinking

This research first focuses on the food insecurity problem among older Canadians. To explain it specifically, food insecurity is considered and examined as an outcome variable as well as a mediator of scholarly interest for two main reasons. First, food insecurity indicates unmet basic food needs facing people in daily lives due to socioeconomic constrains [36]. Respondents are “forced” to experience this constrained life condition. Prior research has argued that food insecurity may negatively affect respondents’ life quality, health, and well-being [36, 37]. Another reason we concentrating on food insecurity is because of data limitation that variables related to eating habits, as crucial health lifestyle indicators, are not usable due to high percentages of missing values and thus they have been excluded. Moreover, the three health lifestyle variables are physical exercise, cigarette smoking, and alcohol drinking.

The main underlying motivation of our scholarly concentration on these variables is because existing studies have argued that they are related to older adults’ health in Canada, the U.S., and many other sociocultural contexts. Specifically, food insecurity may increase the likelihood of people developing mental and physical health problems [38–42]. Similarly, a couple of empirical findings have shown that being physically active benefits older adults’ physical and mental health [43–45]. Physical exercise can also increase older adults’ social participation and engagement [46], which then improves their social health [47]. Smoking, which works in the opposite direction, has been identified as a pivotal predictor of negative health consequences facing older adults, including cancers and all-cause mortality [48–50]. Drinking has also been identified as a risk factor for health [51]. But unlike smoking, it has been argued that drinking can benefit older adults’ social health by providing them with opportunities concerning social engagement. For example, Bareham and colleagues [52] did a systematic review on how older adults perceive their alcohol drinking behavior. Results based on thematic analysis show that many older adults tend to consider themselves as responsible drinkers and drinking plays a role in providing them with chances to engage in social and leisure activities, which can further benefit their health and well-being.

### Research questions

**Fig. 1:**
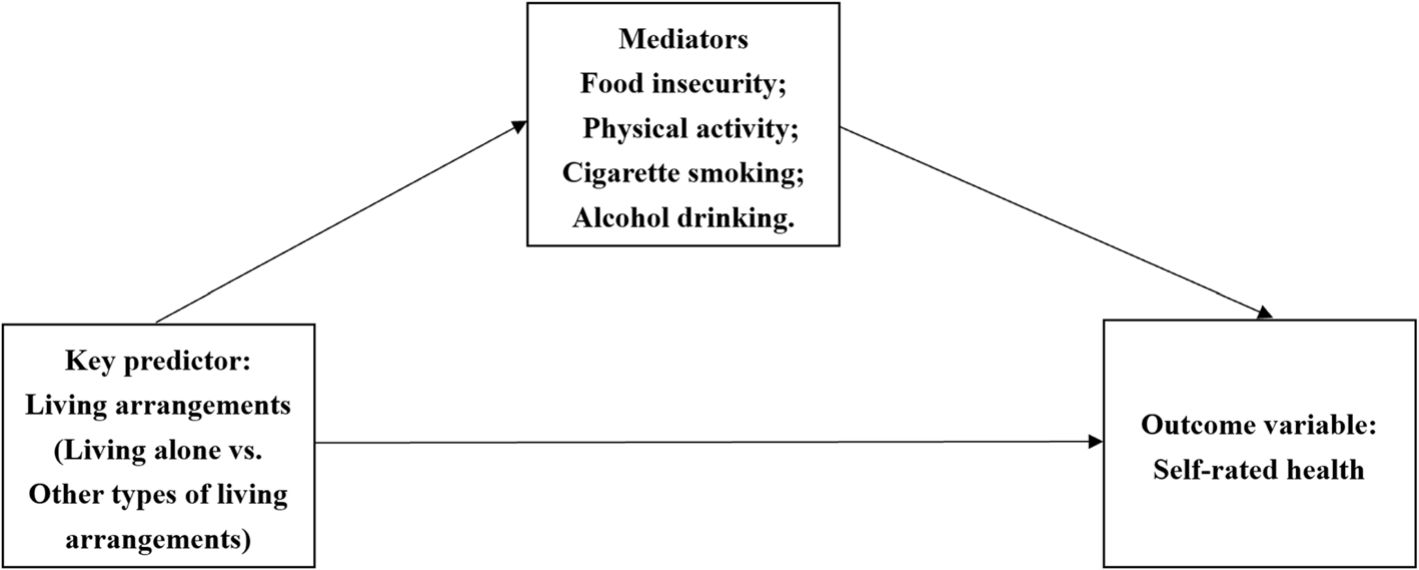
Research framework. *Note*. Covariates that are controlled include respondents’ demographic background, socioeconomic statuses, objective health statuses, and the access to healthcare

The current study aims to explore whether living alone is closely associated with older Canadians’ food insecurity problem, their health lifestyles and self-rated health. Specifically, as shown in Fig. 1, we developed four research questions that are correlated with each other: (1) Compared to other living arrangements, to what extent are older Canadians living alone more or less likely to be confronted with the food insecurity problem and to develop destructive or protective health lifestyles, including physical exercise, smoking, and drinking? (2) To what extent are older Canadians living alone more or less likely to report better self-rated health compared to other living arrangements? (3) To what extent are the food insecurity problem facing older Canadians and their health lifestyles associated with their self-rated health? (4) To what extent can food insecurity and older Canadians’ health lifestyles explain the potential association linking their solo living arrangements and self-rated health?

## Methods

### Data and sample

We used the public version of the 2017–2018 Canadian Community Health Survey (CCHS) to conduct this research. The response rate of the in-use data is 58.8%. Statistics Canada collected and administered the CCHS, and more information is available at: https://www.statcan.gc.ca/en/survey/household/3226.

The 2017–2018 CCHS is the best dataset for this research for two main reasons. First, CCHS covers Canadians aged 12 years and older residing in Canada’s ten provinces and thus is nationally representative. Second, in this dataset, Statistics Canada collected information on older Canadians’ living arrangements, health lifestyles, self-rated health, and a wide range of their demographic, socioeconomic, and health information. Related variables are used as independent and dependent variables, mediators, and controls for statistical analysis. These data characteristics have ensured a smooth modelling process and robust findings for the current study. Also, the missing proportions of these variables are low, further increasing finding reliability and validity.

The overall sample size of the CCHS is 113,290, including 42,575 adults aged 60 and above. The proportion of respondents with missing values is 6.90%; we deleted these respondents directly. The final analytical sample is 39,636.

### Measures

#### The core independent variable

Older Canadians’ living arrangement is used as the core independent variable. The CCHS asked respondent about their “living/family arrangements.” We coded the variable into five categories: living alone, living with spouses/partners, living with spouses/partners and children, single parent living with children only, and other types. We divided the arrangement of living alone into two subcategories based on marriage. The reason is to detect whether older Canadians living apart together are different in health from those living alone but without any intimate relationships. We combined respondents sharing households “with a single parent or two parents with or without siblings” with the “other types” category because of its low weighted percentages in the analytical sample (smaller than 0.5%).

#### Dependent variables and mediators

Five dependent variables have been included: self-reported food insecurity, being physically active or not in the last week, type of smoker, type of drinker, and self-rated health. We also tested food insecurity and the three health lifestyle variables as mediators between older Canadians’ living arrangements and their self-rated health.

The CCHS asked respondents, “[Whether your] food didn’t last and [you have] no money to buy more [in the last] 12 months” We coded the variable into two categories: “never true” and “sometimes true or often true.” As for measuring whether respondents were physically active, we combined three related variables that the CCHS asked, “[Whether] used active transportation [in the last] 7 days,” “[Whether] did sports / fitness / recreational physical activities [in the last] 7 days,” and “[Whether participated in] other physical activities [in the last] 7 days.” All these three variables are dichotomously coded (yes or no) in the original survey. We coded respondents as “0” if they reported participating in none of these physical activities and “1” if they participated in at least one activity. As for types of smoker and drinker, the CCHS asked respondents, “At present, do you smoke cigarettes every day, occasionally or not at all?” and “Type of drinker [in the last] 12 months.” We coded both these two variables into three categories: “Not at all; occasionally; daily” and “Not a drinker; an occasional drinker; a regular drinker.” Worth noting is that regular drinking does not equal to heavy drinking but refer to the frequency of drinking only.

Although some scholars indicate that the validity of self-rated health is to a certain degree moderated by age [53], this way of measuring health reflects people’s understanding of own health status and is still valid in predicting other health outcomes, such as mortality [16, 54–56]. Moreover, Lima-Costa and colleagues [55] pointed out that specific attention should be paid to older adults who report poor health because they often “have accumulated biologic markers of disease” (p.228). We thus agree with Jylhä [57] that self-rated health is a reliable health measure. In the CCHS, respondents were asked: “In general, [how] would you say your health is?” We coded it into three subgroups: “poor or fair,” “good,” and “very good to excellent.” The underlying reason that we coded self-rated health as a three-category variable is because “good” can be different from “very good to excellent” in terms of the level of health status. There is a certain proportion of older Canadians reported their health as “good” rather than higher levels, indicating there exists variability in self-rated health within the aged population. We thus keep “good” as an independent category.

#### Controls

We included five groups of controls that are theoretically associated with older adults’ living arrangements and their health; detailed categorizations of controls are seen in Additional file 1: Appendix 1. We first controlled for respondents’ demographic characteristics, including their age, gender, ethnic background, country of birth, and province of residence. The second group of controls focuses on respondents’ socioeconomic status, including educational attainment, personal income levels, and dwelling ownership.

The third and fourth group focus on older Canadians’ objective health statuses. Specifically, the third group is on older Canadians’ daily mental health status, including mood and anxiety disorders. The fourth group includes current chronic diseases and disability statuses. Chronic condition is based on questions on whether respondents have specific diagnosed chronic diseases, including asthma, arthritis, high blood pressure, heart disease, stroke, diabetes, and cancer. We coded this dichotomously with the two categories: “has no chronic diseases” and “has at least one type of chronic disease.” The set of disability variables capture whether a respondent has at least one type of disability: having difficulties in seeing, hearing, walking, practicing self-care, remembering, and communicating. Related survey questions are based on the Washington Group Disability measure. Respondents were asked, for example, “Do you have difficulty seeing, even if wearing glasses? Would you say [which category listed below suits your situation]?” We coded these variables on specific disability variables as dummies by combining the three categories of having “some difficulty,” having “a lot of difficulties,” and “cannot do at all/unable to do” as one, and the other category is having “no difficulty.”

The last group of controls concerns respondents’ access to healthcare. Two variables have been included, “Has a usual place for immediate care for minor problem” and “Has a regular health care provider.” We coded both as dummies, “no,” and “yes.”

### Analytical modelling

We applied binary logit regressions to predict factors contributing to differences in self-reported food insecurity and being physically active or not, as they are dummy variables. We used ordinal logit regressions to model type of smoker and type of drinkers and self-rated health because they are ordinal variables with three categories each. Results are shown in Odds Ratios (ORs). We presented 95% Confidence Interval (CI) for all ORs. We also ran generalized ordinal logit regressions (gologit) to test the robustness of our results. Moreover, we applied stepwise regressions and the decomposition method for mediation tests. Data analyses were weighted using the probability weight offered by the dataset and conducted using Stata 15.0.

### Formulas

Formula (1) focuses on two dependent dummy variables: food insecurity and physical activity. P_i_ is the probability of having food insecurity (Food insecurity = 1) or being physically active (Physically active = 1). Let x_1_ and β_1_ represent the key independent variable and its coefficient. x_21_, x_22_, … x_2n_ are controls, and β_21_, β_22_, … β_2n_ are the corresponding parameter. Then, x_31_, x_32_, … x_3k_ are mediators, and β_31_, β_32_, … β_3k_ arethe corresponding parameter to be estimated. Moreover, β_0_ is the constant, and ε_0_ is the error term subject to Logit distribution. Formula (1):

$$\ln\left(\frac{P_i}{1-P_i}\right)=\beta_0+\beta_1x_1+\sum_{i=1}^n\beta_{2i}x_{2i}+\sum_{i=1}^k\beta_{3i}x_{3i}+\varepsilon_0$$

Formula (2) is about the other three dependent variables, smoking, drinking, and self-rated health, all of which are ordinal variables. P(y ≤ j) is the cumulative probability of dependent variables less than or equal to a specific category j = 1, 2, 3. x_1_ and β_1_ also represent the key independent variable and its coefficient. x_21_, x_22_, … x_2n_ and x_31_, x_32_, … x_3k_ represent controls and mediators respectively. β_21_, β_22_, … β_2n_ and β_31_, β_32_, … β_3k_ are their corresponding parameter. Moreover, β_j_ (j = 1, 2, 3) is the constant, and ε_j_ (j = 1, 2, 3) is the error term subject to ordinal Logit distribution. Formula (2):

$$\mathit{\ln}\left(\frac{P\ \left(y\leqslant j\right)}{1-P\left(y\leqslant j\right)}\right)={\beta}_j+{\beta}_1{x}_1+\sum_{i=1}^n{\beta}_{2i}{x}_{2i}+\sum_{i=1}^k{\beta}_{3i}{x}_{3i}+{\varepsilon}_j$$

## Results

**Fig. 2:**
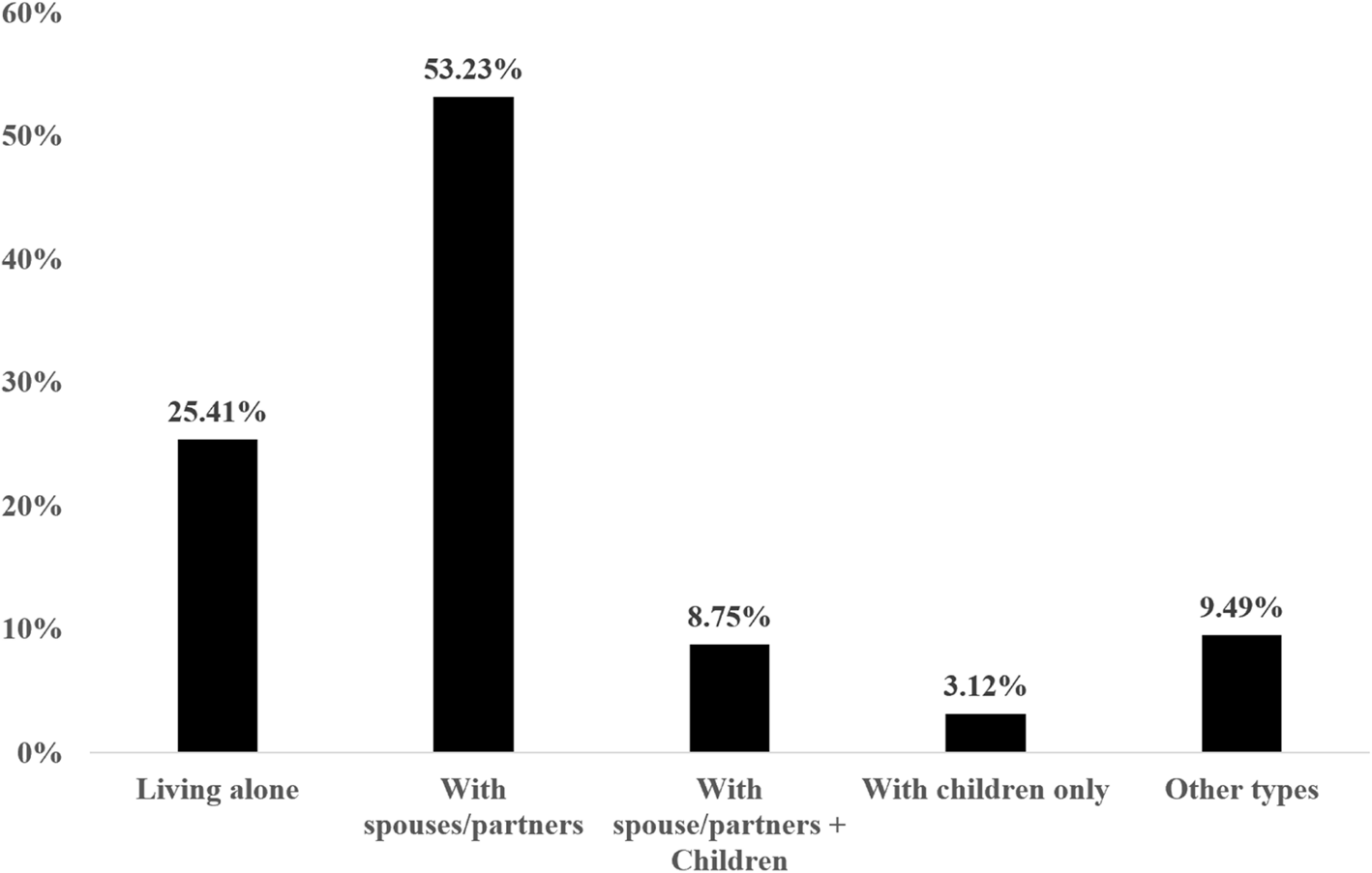
Weighted living arrangements (%) of Canadians aged 60 and above

Figure 2 shows weighted percentages of living arrangements among older Canadians aged 60 and above. As presented, the most prevalent living arrangement among older Canadians is living with spouses/partners only (53.23%). Meanwhile, 25.41% of older Canadians live alone, which is the second highest among all living arrangements. Also, there are 8.75% of respondents living with both spouses/partners and children, and 3.12% living with children only. There are also 9.49% of respondents who reported other types of living arrangements, but it is unclear their actual statuses due to data limitation.

Table 1 shows the weighted characteristics of the analytical sample by their living arrangements. Results show bivariate relationships that the living arrangement variable is associated with food insecurity, health lifestyles, and self-rated health (*p* < 0.001). In particular, older Canadians living alone have a higher proportion reporting food insecurity issue (6.86%) compared to those living with spouses/partners and with children (2.86%) or without children (1.95%). But the percentage is lower than those living with children only (9.04%). Similarly, older Canadians living alone perform better in physical exercise than those living with children (83.57% vs. 76.90%) but the percentage is lower compared to those living with spouses/partners (87.62%) and those living with both spouses/partners and children (86.46%). As for smoking, living alone is associated with the highest proportion of smoking daily (12.88%) among all living arrangements except living with others (14.04%). The proportion of drinking regularly of those living alone stays in the middle (51.93%), which is lower than those living with spouses/partners (65.06%) and those living with both (57.36%) but a lot higher compared to those living with children only (41.76%). Lastly, living alone is correlated with poor/fair self-rated health (21.27%) in comparison to living with spouses/partners with children (16.52%) or without children (16.10%). However, those living with children only is a bit higher regarding the percentage of reporting poor/fair health (24.52%) compared to their counterparts living alone. To sum up, bivariate associations indicate that there exist some differences in food insecurity, health lifestyles and self-rated health between solo-living older Canadians and their co-residing counterparts.

**Table 1:** Weighted Percentages (%) of living arrangements, food insecurity, physical activity, type of smoker, type of drinker, and self-rated health of the analytical sample, *N* = 39,636, Canadian Community Health Survey 2017–2018 Annual Component

	Living alone	Living with spouses/partners	Living with spouses/partners + children	Living with children	Other types	Design-based F statistic
***Food didn’t last and no money to buy more***						***
Never true	**93.14**	**98.05**	**97.14**	**90.96**	**93.06**	
Sometimes true / Often true	**6.86**	**1.95**	**2.86**	**9.04**	**6.94**	
***Being physically active or not***						***
No	**16.43**	**12.38**	**13.54**	**23.10**	**17.24**	
Yes	**83.57**	**87.62**	**86.46**	**76.90**	**82.76**	
***Type of smoker***						***
Not at all	**84.20**	**90.89**	**88.73**	**84.67**	**84.14**	
Occasionally	**2.91**	**1.86**	**2.54**	**3.52**	**1.81**	
Daily	**12.88**	**7.25**	**8.73**	**11.82**	**14.04**	
***Type of drinker in the last 12 months***						***
Did not drink in the last 12 months	**29.77**	**20.94**	**25.69**	**36.13**	**42.08**	
Occasional drinker	**18.31**	**14.00**	**16.95**	**22.11**	**20.61**	
Regular drinker	**51.93**	**65.06**	**57.36**	**41.76**	**37.31**	
***Self-rated physical health***						***
Poor/Fair	**21.27**	**16.10**	**16.52**	**24.52**	**21.63**	
Good	**32.10**	**30.36**	**35.94**	**35.44**	**38.09**	
Very good to excellent	**46.62**	**53.54**	**47.54**	**40.04**	**40.28**	

*Note*. * *p* < 0.05; ** *p* < 0.01; *** *p* < 0.001

Table 2 presents odds ratios from weighted logistic regressions predicting self-reported food insecurity and physical activity among older Canadians. Models 1a and 2a controls for respondents’ demographic backgrounds, socioeconomic statuses, objective health statuses, and their access to healthcare. Model 1b further controls for all health lifestyle variables, and Model 2b additionally controls for the other two health lifestyles and food insecurity.

**Table 2:** Odds ratios and 95% CI from weighted logistic regression models of self-reported food insecurity and physical activity among Canadians aged 60+, N = 39,636, Canadian Community Health Survey 2017–2018 Annual Component

	Self-reported food insecurity		Physical activity	
	Model 1a	Model 1b	Model 2a	Model 2b
***Living arrangements (Living alone)***				
With spouses/partners	0.36 [0.28, 0.46]***	0.38 [0.30, 0.49]***	**0.96 [0.87, 1.07]**	**0.91 [0.82, 1.02]**
With spouses/partners and children	0.35 [0.22, 0.56]***	0.36 [0.22, 0.58]***	**0.81 [0.61, 1.07]**	**0.79 [0.59, 1.04]**
With children only	**1.13 [0.75, 1.71]**	**1.11 [0.73, 1.68]**	**0.77 [0.55, 1.07]**	**0.77 [0.55, 1.06]**
Other	**0.67 [0.45, 1.01]**	0.66 [0.44, 0.98]*	**0.95 [0.76, 1.18]**	**0.98 [0.78, 1.21]**
***Self-reported food insecurity (Never true)***				
Sometime true / Often true				**1.09 [0.85, 1.38]**
***Being physically active or not (No)***				
Yes		**0.98 [0.78, 1.23]**		
***Type of smoker (Not at all)***				
Occasionally smoke		2.48 [1.67, 3.68]***		**0.93 [0.64, 1.35]**
Daily smoke		1.81 [1.45, 2.27]***		0.70 [0.59, 0.83]***
***Type of drinker (Did not drink at all)***				
Occasionally drink		**0.86 [0.66, 1.13]**		1.35 [1.17, 1.55]***
Regularly drink		0.65 [0.52, 0.81]***		1.72 [1.53, 1.94]***
***Pesudo R***^***2***^	**0.2144**	**0.2240**	**0.1239**	**0.1319**

*Note*. * *p* < 0.05; ** *p* < 0.01; *** *p* < 0.001. Models 1a and 2a control for all five sets of covariates: respondents’ demographic backgrounds, socioeconomic statuses, mental health statuses, chronic diseases and disability conditions, and older adults’ access to healthcare. Model 1b controls for all five sets of covariates and all three health lifestyle variables. Model 2b controls for all five sets of covariates, food insecurity, and the other two health lifestyle variables

As shown in Model 1a, compared to those living alone, respondents living with spouses/partners are much less likely to exposure to the risk of food insecurity (OR 0.36, 95% CI 0.28–0.46, *p* < 0.001); so are those living with both spouses/partners and children (OR 0.35, 95% CI 0.22–0.56, *p* < 0.001). Such a significant association does not vary in Model 1b that the odds of those living with spouses/partners only is about 60% lower in terms of exposing to food insecurity risks (OR 0.38, 95% CI 0.30–0.49, *p* < 0.001). The odds of facing the food insecurity problem among those living with both is also more than 60% lower compared to that among who living solely (OR 0.36, 95% CI 0.22–0.58, *p* < 0.001). Older Canadians living in other types of arrangements are also less likely to face the food insecurity issue (OR 0.66, 95% CI 0.44–0.98, *p* < 0.05).

Models 2a and 2b present the association between living arrangements and physical activity. Results of both models indicate that older Canadians living alone are not significantly different from their counterparts co-residing with families in terms of exercising in daily lives, net of all covariates.

The likelihoods of cigarette smoking and alcohol drinking of older Canadians living alone, especially compared to their counterparts living with families, are presented in Table 3. Like the modelling shown in Table 2, Models 1a controls for all covariates, and Models 1b further adds the other two health lifestyle variables and food insecurity. The logic remains the same for Models 2a and 2b.

**Table 3:** Odds ratios and 95% CI from weighted logistic regression models of type of smoker and type of drinker among Canadians aged 60+, N = 39,636, Canadian Community Health Survey 2017–2018 Annual Component

	Type of smoker		Type of drinker	
	Model 1a	Model 1b	Model 2a	Model 2b
***Living arrangements (Living alone)***				
With spouses/partners	0.54 [0.48, 0.61]***	0.56 [0.49, 0.63]***	1.43 [1.33, 1.54]***	1.43 [1.32, 1.54]***
With spouses/partners and children	0.66 [0.51, 0.85]**	0.68 [0.52, 0.88]**	**1.09 [0.92, 1.29]**	**1.09 [0.92, 1.28]**
With children only	**1.04 [0.69, 1.59]**	**1.01 [0.66, 1.55]**	**0.91 [0.73, 1.14]**	**0.93 [0.75, 1.16]**
Other	**1.04 [0.83, 1.30]**	**1.06 [0.85, 1.32]**	0.79 [0.69, 0.92]**	0.79 [0.68, 0.92]**
***Self-reported food insecurity (Never true)***				
Sometime true / Often true		1.67 [1.36, 2.04]***		0.73 [0.61, 0.88]***
***Being physically active or not (No)***				
Yes		0.75 [0.64, 0.87]***		1.56 [1.41, 1.71]***
***Type of smoker (Not at all)***				
Occasionally smoke				1.37 [1.09, 1.72]**
Daily smoke				**0.98 [0.87, 1.11]**
***Type of drinker (Did not drink at all)***				
Occasionally drink		**1.13 [0.96, 1.32]**		
Regularly drink		**1.09 [0.95, 1.25]**		
***Pseudo R***^***2***^	**0.0905**	**0.0935**	**0.0958**	**0.0993**
***Cut point 1***	**−0.29**	**−0.42**	**−0.87**	**−0.50**
***Cut point 2***	**−0.04**	**−0.16**	**−0.01**	**0.36**

*Note*. * *p* < 0.05; ** *p* < 0.01; *** *p* < 0.001. Models 1a and 2a control for five sets of covariates: respondents’ demographic backgrounds, socioeconomic statuses, mental health statuses, chronic diseases and disability conditions, and their access to healthcare. Models 1b and 2b control for all five groups of covariates, food insecurity, and the other two health lifestyle variables

Model 1a reveals that living with spouses/partners is significantly associated with lower frequencies of smoking compared to living alone (OR 0.54, 95% CI 0.48–0.61, *p* < 0.001), and this association remains in Model 1b (OR 0.56, 95% CI 0.49–0.63, *P* < 0.001). Similarly, the odds of older Canadians living with both spouses/partners and children having smoking issues are more than 30% lower compared their counterparts living alone, as indicated by Model 1a (OR 0.66, 95% CI 0.51–0.85, *p* < 0.01) and 1b (OR 0.68, 95% CI 0.52–0.88, *p* < 0.01). Living with children only or living in other kinds of arrangements are not significantly different from living alone in smoking.

As for drinking, results of both Models 2a and 2b indicate that respondents living with spouses/partners are about 43% (OR 1.43) more likely to have higher frequencies of drinking compared to those living alone (*p* < 0.001). The 95% CI in Model 2a is 1.33 to 1.54 and in Model 2b is 1.32 to 1.54. In contrast, living in other types of arrangements is significantly associated with lower odds of drinking compared to living alone (OR 0.79, 95% CI 0.68–0.92, *p* < 0.01), as shown in Model 2b. Moreover, older Canadians living alone are not different from their counterparts who live with children with or without spouses/partners in terms of drinking alcohol.

Table 4 presents factors contributing to older Canadians’ self-rated health. In Model 1, respondents’ demographic backgrounds, socioeconomic statuses, objective health statuses, and the access to healthcare have been controlled. Results show that older Canadians living with spouses/partners have reported better health compared to those living alone (OR 1.12, 95% CI 1.03–1.20, *p* = 0.004), but the health disparity between the two living arrangements is relatively small. But there exists no difference in self-rated health between living alone and other three living arrangements.

**Table 4:** Odds ratios and 95% CI from weighted ordinal logistic regression models of whether Self-reported food insecurity, being physically active, type of smoker, and type of drinker medicate the association between living arrangements and self-rated health among Canadians aged 60+, N = 39,636, Canadian Community Health Survey 2017–2018 Annual Component

	Self-rated Health				
	Model 1	Model 2	Model 3	Model 4	Model 5
***Living arrangements (Living alone)***					
With spouses/partners	1.12 [1.03, 1.20]**	1.10 [1.02, 1.18]*	1.08 [1.00, 1.17]*	**1.08 [1.00, 1.16]**	**1.03 [0.96, 1.12]**
With spouses/partners and children	**0.96 [0.82, 1.13]**	**0.94 [0.80, 1.11]**	**0.94 [0.80, 1.10]**	**0.96 [0.81, 1.12]**	**0.94 [0.80, 1.10]**
With children only	**1.04 [0.82, 1.31]**	**1.04 [0.83, 1.32]**	**1.05 [0.82, 1.33]**	**1.06 [0.85, 1.33]**	**1.11 [0.88, 1.39]**
Other	**1.11 [0.94, 1.30]**	**1.10 [0.93, 1.29]**	**1.11 [0.94, 1.30]**	**1.14 [0.97, 1.34]**	**1.14 [0.97, 1.33]**
***Self-reported food insecurity (Never true)***					
Sometime true / Often true		0.63 [0.52, 0.77]***			0.68 [0.56, 0.82]***
***Type of smoker (Not at all)***					
Occasionally smoke			0.67 [0.54, 0.83]***		0.66 [0.53, 0.83]***
Daily smoke			0.58 [0.52, 0.66]***		0.60 [0.53, 0.68]***
***Type of drinker (Did not drink at all)***					
Occasionally drink				**1.10 [0.99, 1.22]**	**1.07 [0.96, 1.19]**
Regularly drink				1.64 [1.50, 1.78]***	1.57 [1.44, 1.71]***
***Being physically active or not (No)***					
Yes					1.93 [1.75, 2.13]***
***Pseudo R***^***2***^	**0.1655**	**0.1664**	**0.1687**	**0.1707**	**0.1801**
***Cut point 1***	**−2.44**	**−2.49**	**−2.65**	**−2.19**	**−1.90**
***Cut point 2***	**−0.42**	**−0.47**	**−0.63**	**−0.16**	**0.17**

*Note*. * *p* < 0.05; ** *p* < 0.01; *** *p* < 0.001. Model 1 controls for respondents’ demographic backgrounds, socioeconomic statuses, objective health conditions, and their access to healthcare. On top of that, Models 2 to 4 further control for food insecurity, smoking, and drinking, respectively. Model 5 controls for all covariates, food insecurity, and health lifestyle variables

Models 2 to 4 are stepwise regressions to test whether food insecurity and health lifestyles mediate the association between respondents’ living arrangements and self-rated health. Being physically active or not is excluded in mediation test because living alone is uncorrelated with it. Results show that the odds ratios decrease and *p* values increase after adding food insecurity (OR 1.10, 95% CI 1.02–1.18, *p* = 0.017), cigarette smoking (OR 1.08, 95% CI 1.00–1.17, *p* = 0.042), and alcohol drinking (OR 1.08, 95% CI 1.00–1.16, *p* = 0.058). These value changes indicate there may exist mediation effects. We further explored the magnitudes of the mediation effects using the decomposition method. Results show that, although the values of indirect effects are relatively small, 32.20%, 50.20%, and 48.00% of the total effects regarding the association between living arrangements (living alone vs. living with spouses/partners) and self-rated health can be explained by food insecurity, cigarette smoking, and alcohol drinking, respectively. Results of Sobel tests are all significant (*p* < 0.001). Moreover, if objective health indicators were not controlled in the models due to the reason that they may account for some variabilities in self-rated health, the mediation effects of the three health lifestyles remain with the value of 21.60% (food insecurity), 21.20% (smoking), and 20.50% (drinking). The results have also passed Sobel tests (*p* < 0.001). These results confirm that the three health lifestyle variables partially explain why older Canadians living alone have reported lower levels of health compared to those living with spouses/partners.

Additionally, as Model 5 presents, food insecurity and all health lifestyles are significantly associated with self-rated health. In particular, the odds of reporting better self-rated health among those who face insecure food supply in daily lives are about 30% lower compared to their counterparts who have no food insecurity issue (OR 0.68, 95% CI 0.56–0.82, *p* < 0.001). Similarly, older Canadians smoking occasionally (OR 0.66, 95% CI 0.53–0.83) or daily (OR 0.60, 95% CI 0.53–0.68) are less likely to report better self-rated health with a statistical significance (*p* < 0.001). In contrast, regular drinking is associated with about 60% more likely to have better self-rated health (OR 1.57, 95% CI 1.44–1.71, *p* < 0.001). The odds of reporting better self-rated health among respondents who are physically active are about two times more compared to those who are not (OR 1.93, 95% CI 1.75–2.13, *p* < 0.001).

### Robustness checks

We ran robustness checks for the above-listed multivariate regression models. We applied the generalized ordinal logit regressions (gologit) to avoid possible violations against parallel assumptions [58, 59]. That is to say, a leading advantage of gologit over ologit is that the former can provide more detailed comparisons between categories of an ordinal dependent variable based on differences in the core independent variable of researchers’ interest [58]. For example, for each ordinal dependent variable in our research, gologit presented us two sets of odds ratios of living arrangements predicting, first, poor/fair health (health = 1) over good plus very good to excellent health (health = 2, 3), and second, poor/fair health plus good health (health = 1, 2) over very good to excellent health (health = 3).

The gologit results are close to what we have based on ologit regarding magnitudes and significance levels of odds ratios. One place needs to report is that older Canadians living with spouses/partners still have higher odds of reporting very good to excellent health compared to their solo-living counterparts (OR 1.09, 95% CI 1.00–1.18, *p* = 0.047), controlling for all five sets of covariates and alcohol drinking. We think it would remotely affect the robustness of our results for two reasons. First, the level of this statistical significance is relatively low. Another reason is that, according to the gologit results, the odds of older Canadians reporting good health or above over poor/fair health due only to the difference between living alone versus living with spouses/partners is insignificant (*p* = 0.387), net of all controls. This consistency confirms the robustness of our results. Results of robustness checks are available upon request.

## Discussions

In this research, we explored living alone as a social determinant of health among older Canadians and further tested to what extent food insecurity and health lifestyles as possible mediators that can explain the association between living alone and self-rated health. Our findings first revealed that solo-living older Canadians differ in health lifestyles from those living with spouses/partners with or without children. This demonstrates that living arrangements work as a crucial type of collectivity that can mold people’s health lifestyles, which is a theoretical contribution to Cockerham’s theory. Four mediators have been examined: self-reported food insecurity and three health lifestyle variables, including physical exercise, frequencies of smoking and drinking. According to our results, compared to those living with spouses/partners, living alone is also significantly related to more insecure situations in terms of getting access to food and worse health lifestyles, including smoking more often, and having fewer opportunities to drink for older Canadians. Similarly, compared to those living with spouses/partners and children, older Canadians who lived alone were more likely to be confronted with food insecurity issues; also, they had higher odds of being a daily smoker. But living arrangements do not significantly affect the participation in physical activities for older Canadians, which is in line with a recent study’s [12] findings that older Canadians living alone spent a comparable amount of time participating in physical activities compared to counterparts living with spouses/partners with or without children. One point that should be noticed is that the arrangement of living with children only may be due to the fact that some older adults have relatively unhealthier conditions, which may have further affected their physical activity.

Our findings have further revealed a significant relationship between living arrangements and older Canadians’ self-rated health, and to what extent their health lifestyles account for the relationship. Specifically, in line with prior studies in some other societies [3, 18, 44], older Canadians who lived with spouses/partners reported better physical health compared to their living alone counterparts, although the difference is relatively small after controlling for all covariates. This indicates that older Canadians living by themselves are confronted with structured life options, under which they may have developed harmful health lifestyles. Further, we found mediation effects of food insecurity and two health lifestyles, including cigarette smoking and alcohol drinking, that can partially explain why solo-living older Canadians have lower self-rated health statuses compared to their counterparts living with spouses/partners. In addition, food insecurity, smoking, drinking, and being physically active are significantly associated with respondents’ self-rated health. These results warrant the importance of guaranteeing secure food supply and developing healthier lifestyles for older Canadians. This may especially be necessary for older adults living alone because they reported worse performance in some health lifestyles compared to those living with spouses/partners or with both spouses/partners and children.

The theoretical and policy-oriented connotations of these empirical findings clearly show that food insecurity and health lifestyles are closely associated with older adults’ health. Our results support prior findings demonstrating the linkages between food insecurity [39, 60] or smoking [48, 50, 61] and older adults’ health. Specifically, although the proportion of older Canadians facing food insecurity is relatively low (3.97% in our research), existing research has illustrated the harmful effects of food insecurity on older adults’ health [37, 39]. Also, the proportion of Canadians aged 65 and above who smoked daily or occasionally was 8.4% in 2020 and 8.7% in 2021 [62], highlighting the importance of exploring older smokers’ health. Therefore, local communities and health officials are suggested to pay more attention to food insecurity and heavy smoking issues among older Canadians especially those who live by themselves because they are likely to face these health-lifestyle-related problems due to fewer life choices. Specifically, older Canadians who lack financial support and live in poverty are suggested to be prioritized because living alone, poverty, and unhealthy lifestyles are often correlated and, thus, are clustered [36].

Health implications of alcohol drinking are complex. Medical studies have demonstrated that drinking alcohol has no positive health consequences [51]. A systematic review done by Di Castelnuovo et al. [63] has revealed a more complicated association between drinking and health that below a critical point drinking plays a protective role but above the critical point overdoses of drinking are associated with increased mortality. In comparison, our findings show the positive importance of occasional or regular drinking for older Canadians’ health. To note, regular drinking is only about the frequency of drinking. In the survey, the information regarding the dose of drinking is accessible that most of those who self-reported as regular or occasional drinkers never drank 5 (male) or 4 (female) or more drinks on one occasion in the past year, or the frequency can be low (less than once a month). This indicates that older Canadians may probably drink for the purpose of social involvement and regular drinking does not equal to heavy drinking at all. Therefore, our findings are only on the societal dimension of drinking rather than offering any medical evidence; importantly, the potential harmfulness of drinking to older adults’ health should not be ignored. Certainly, we suggest further explorations concerning the association between alcohol drinking and older adults’ health by concentrating on what detailed mechanisms can possibly work in between. Supposing that it is because alcohol drinking provides older adults with opportunities to participate in social activities for better social engagement [53, 64], in that case, communities can work more on this to increase older Canadians’ social engagement [65]. Plenty of health-benefiting techniques work efficiently to help them avoid isolation, such as establishing social connections sites [66] and promoting the application of information and communication technologies [67, 68], especially under the ongoing COVID-19 pandemic [69].

A final point that is worth noting focuses on the fact that no significant difference exists in food insecurity or health lifestyles and self-rated health between older adults who lived alone but were in marriage/partnership during the survey time (about 1%) and those purely living alone. The former can be seen a specific type of living apart together. In Western countries, such as Canada [70], the Netherlands [71], and the U.S. [72], living apart together has been increasingly practiced among older adults. But our findings reveal that co-residing with intimate ones works more positively to older adults’ health lifestyles (e.g., less smoking) compared to this important category of living apart together. Results are upon further request.

### Limitations

Four research limitations should be noticed. First, no causality has been identified in our research. We only explored the associations between living arrangements (living alone vs. other types of living arrangements) and the food insecurity problem facing older Canadians, their health lifestyles as well as self-rated health due mainly to the fact that the in-use dataset is cross-sectional. However, there may exist two-way causality between living alone and self-rated health and between some health lifestyles (e.g., being physically active, drinking) and self-rated health. Future studies are suggested to detect the causality by addressing how the durations and transitions of older adults’ living arrangements and health lifestyles may have affected their health from a longitudinal perspective. Second, we only focused on food insecurity and three types of health lifestyles, mainly because of data limitations. Scholars should consider more health lifestyles, such as eating habits (e.g., vegetable, fruit, and meat intake) and sleeping, to detect more possible underlying mechanisms linking living alone and health consequences among older adults in Canada and beyond. Third, we did not explore older Canadians living in institutions and whether there are differences in their health compared to those living alone. This is because we lack related data in this regard due to the fact that CCHS data do not cover respondents living in institutions. Future work can focus on this part under Canada’s population aging context. Lastly, it remains unclear who those “living with others” are, and therefore, we did not systematically discuss the differences between living alone and living with others in the current research. Despite these limitations, our research is among the very few ones applying health lifestyle theory to identify possible contributions of living arrangements (especially the comparisons between living alone vs. living with spouses/partners with or without children) to older adults’ health and the underlying mechanisms amid this chain.

## Conclusion

Although many scholarly endeavors have explored living alone as a health predictor, especially for the aged, attention has rarely been paid to whether older adults living alone report better or worse health lifestyles. We applied Cockerham’s health lifestyle theory in this research and used the CCHS 2017–2018 Annual Component data to identify the magnitude to which living alone is associated with older Canadians’ insecure food supply problem and their health lifestyles. The health lifestyle theory emphasizes the mechanisms of how people develop their health-related lifestyles through the interactions between life choices (agency) and life chances (structure) [31, 33]. Since living arrangements matter to how older adults handle emotion, needs of care, interactions and communications, and other aspects of daily lives, we consider it a potential collective-level factor to older adults’ health lifestyles.

In this research, we included food insecurity and three health lifestyles, namely physical exercise, cigarette smoking, and alcohol drinking, which may be crucial to older adults’ health. Based on our findings, there are three crucial take-home messages. First, compared to those living within intimate relationships, older adults living alone are more likely to have food insecurity problems, heavy smoking issues, and fewer chances to socially drink. The most notable difference is between older adults living alone and their counterparts living with spouses/partners. In addition, our results also indicate that older adults living alone reported worse self-rated health than those living with spouses/partners. This health disparity, although not large, can be partly explained by food insecurity, smoking, or drinking, respectively. Lastly, food insecurity and all three health lifestyles of interest are closely related to older Canadians’ self-rated health with statistical significance. These findings and messages entail specific attention to solo-living older Canadians.

Our results have passed the robustness check, further indicating their validity and generalizability to the entire older Canadian population and, therefore, providing a basis for future policymaking. Because we did not take more health lifestyles into consideration due mainly to data limitations, more scholarly explorations regarding this matter are recommended.

## Author notes

1. The coverage of the 2017–2018 CCHS excludes “persons living on reserves and other Aboriginal settlements in the provinces; full-time members of the Canadian Forces; the institutionalized population, children aged 12-17 that are living in foster care, and persons living in the Quebec health regions of Région du Nunavik and Région des Terres-Cries-de-la-Baie-James.” [73]. But these exceptions only occupy a remotely small proportion of the Canadian population aged 12 and above. Link: https://www23.statcan.gc.ca/imdb/p2SV.pl?Function=getSurvey&Id=329241
2. Regarding the measure of chronic disease variable, diseases such as chronic bronchitis, emphysema or chronic obstructive pulmonary disease, high blood cholesterol or lipids, and urinary incontinence were skipped in the CCHS 2017–2018 Annual Component because they were included in previous CCHS data.
3. There are also many covariates that have been controlled are significantly associated with health lifestyles and self-rated health (see notes 4-8). Detailed results are not shown because of space limitation but are available based on requests.
4. *Food insecurity*. Age groups, racial background, immigrant status, province of residence, personal income, dwelling ownership, mood disorder, having disabilities in seeing, hearing, walking, self-care, and remembering, smoking, and drinking are significantly associated with have food insecurity issues facing the respondents.
5. *Physical activity*. Age groups, province of residence, gender, educational attainment, dwelling ownership, mood disorder, having chronic diseases, having disabilities in walking and self-care, smoking, and drinking are significantly associated with respondents’ participation in physical activities.
6. *Cigarette smoking*. Age groups, racial background, immigrant status, province of residence, gender, educational attainment, personal income, dwelling ownership, having mood and anxiety disorder, having disabilities in walking and remembering, the access to regular health provider, food insecurity, and being physically active are significantly associated with cigarette smoking among older Canadians.
7. *Alcohol drinking*. Age groups, racial background, immigrant status, province of residence, gender, educational attainment, personal income, dwelling ownership, having mood disorder, having disabilities in walking, self-care, and communicating, the access to immediate care for minor health problems, food insecurity, being physically active, and smoking are significantly associated with alcohol drinking among older Canadians.
8. *Self-rated health*. Age groups, racial background, immigrant status, province of residence, gender, educational attainment, personal income, dwelling ownership, having mood and anxiety disorder, having chronic diseases, having disabilities in all kinds, food insecurity, smoking, drinking, and being physically active are significantly associated with older Canadians’ self-rated health.
9. We have also taken the health differences between the young-old (aged 60–69) and middle-oldest old (aged 70 and above) into account. The aged population is not homogeneous. Existing studies divide older adults into two or three groups with different terminologies. For example, dichotomous categorizations include “young-old” and “old-old” [74–79], or “young-old” and “older adults” [80]. Different tripartitions include “young-old,” “old-old,” and “oldest old” [81], “young-old,” “the-old,” and “old-old” [82], “young-old,” “middle-old,” and “old-old” [83], or “young-old,” “middle-old,” and “oldest old” [84], and so forth.

Although no agreement on the age thresholds of these categorizations has been reached, many prior studies have indicated that stratified age groups vary in health behaviors they maintain [85], health issues they face, and health needs they concern [75], and health statuses they have [74]. For instance, Zavagnin and colleagues [79] revealed the difference in cognitive decline between the young-old and the old-old. Additionally, Gouveia and associates [74] explored related factors contributing to Health-Related Quality of Life (HRQoL) among older adults residing in Madeira, Portugal. Researchers found that physical activity is positively associated with older adults’ HRQoL. Such an association is significantly more prominent among those old-old adults aged 70 and above than the young-old aged between 60 and 69, indicating the importance of increasing physical activity among the old-old. Similarly, Liu et al. [23] found that age is significantly related to HRQoL among older Chinese living by themselves, with the more aged scoring lower on HRQoL. Another study indicates that old-old Koreans – defined as those aged 75 and above – face a greater likelihood of having impairments in activities of daily living compared to those aged between 65 and 74 [75]. Besides the age effect that health decline is inevitable during personal aging, there also exist cohort effects that may contribute to health disparities across age groups. In the 1970s, Neugarten’s [77] research on age groups in the U.S. revealed that the rising young-old cohort would be much more educated than the old-old cohort. Historical events may also have contributed to the cohort effect. Krause and colleagues [81] figured out that traumatic experiences in early life course stages may have negatively affected the young-old cohort of Americans aged 65–74 years in 1992, the baseline survey time, the most due to the economic upturn following the Second World War “may make severe adversity more difficult for members of the young-old cohort to tolerate.” (p.645–646).

Older adults’ living arrangements also differ between age-based cohorts in that the young-old are significantly less likely to live alone than their older counterparts [74]. In Canada, for instance, although the gender gap in life expectancy has decreased, losing partners/spouses may be the main underlying reason for the disparity in older adults living alone [1]. Moreover, compared to the young-old, those older ones may be more likely to live with others or live in institutions because of the increased need for timely healthcare due to health decline.

Because of these above-mentioned differences in living arrangements and health outcomes due to age, we divided older Canadians into two subpopulations in this research. We define the “young-old” as those between 60 and 69 years at the time of the survey and the “middle-oldest old” as those aged 70 years and above to detect the mediation effect of health lifestyles in the potential association between living arrangements and physical health. But we found that the association patterns of living arrangements with food insecurity, the three health lifestyles (being physically active, smoking, drinking), as well as self-rated health are quite similar between the young-old and the middle-oldest old. We thus did not present the two subpopulations separately. Results are upon requests.

## Supplementary Information

**Additional file 1: Appendix 1.** Weighted characteristics of the analytical sample, N=39,636, Canadian Community Health Survey 2017-2018 Annual Component.

## Data Availability

The dataset used and analyzed during the current study is the public-use version of the 2017–2018 Canadian Community Health Survey Annual Component. The dataset is available for public use. The dataset is available from the corresponding author upon reasonable requests.
