# Supplementary material for: Investigating food insecurity, health lifestyles, and self-rated health of older Canadians living alone
Source: BMC Public Health. 2022 Dec 5;22:2264. doi: 10.1186/s12889-022-14467-0 (PMC9720941; doi:10.1186/s12889-022-14467-0)
Supplement: Supplementary file 1 — Additional file 1: Appendix 1. Weighted characteristics of the analytical sample, N=39,636, Canadian Community Health Survey 2017-2018 Annual Component. [file 12889_2022_14467_MOESM1_ESM.docx]

**Appendix 1. Weighted characteristics of the analytical sample, N=39,636, Canadian Community Health Survey 2017-2018 Annual Component.**

|  | **Living alone** | **Living with spouses/partners** | **Living with spouses/partners + children** | **Living with children** | **Other types** | **Design-based F statistic** |
| --- | --- | --- | --- | --- | --- | --- |
| ***Age groups*** |  |  |  |  |  | ******* |
| 60-64 | **23.41** | **28.74** | **51.53** | **28.44** | **31.44** |  |
| 65-69 | **21.55** | **26.32** | **23.01** | **18.99** | **25.33** |  |
| 70-74 | **18.07** | **21.31** | **12.88** | **12.43** | **16.45** |  |
| 75-79 | **14.07** | **13.15** | **6.16** | **9.56** | **11.75** |  |
| 80+ | **22.90** | **10.47** | **6.42** | **30.58** | **15.04** |  |
| ***Gender*** |  |  |  |  |  | ******* |
| Male | **34.45** | **53.63** | **59.38** | **24.26** | **40.19** |  |
| Female | **65.55** | **46.37** | **40.62** | **75.74** | **59.81** |  |
| ***Racial background*** |  |  |  |  |  | ******* |
| Non-minorities | **90.59** | **89.64** | **72.29** | **81.38** | **63.78** |  |
| Minorities | **9.41** | **10.36** | **27.71** | **18.62** | **36.22** |  |
| ***Country of birth*** |  |  |  |  |  | ******* |
| Canadian born | **80.47** | **78.10** | **58.53** | **65.94** | **56.06** |  |
| Landed immigrant | **19.53** | **21.90** | **41.47** | **34.06** | **43.94** |  |
| ***Province of residence*** |  |  |  |  |  | ******* |
| Ontario | **34.82** | **35.36** | **48.36** | **44.91** | **45.34** |  |
| Quebec | **29.50** | **26.10** | **17.40** | **22.93** | **15.83** |  |
| BC | **13.65** | **13.44** | **14.41** | **13.27** | **15.57** |  |
| Alberta | **8.19** | **9.69** | **9.43** | **7.80** | **10.99** |  |
| Other provinces | **13.84** | **15.40** | **10.40** | **11.10** | **12.28** |  |

**Appendix 1 continued.**

| ***Educational attainment*** |  |  |  |  |  | ******* |
| --- | --- | --- | --- | --- | --- | --- |
| Elementary school and below | **25.00** | **18.04** | **17.27** | **30.00** | **30.36** |  |
| Secondary and high school | **23.61** | **24.49** | **21.45** | **23.37** | **23.52** |  |
| College and above | **51.39** | **57.47** | **61.28** | **46.64** | **46.12** |  |
| ***Personal income*** |  |  |  |  |  | ******* |
| <20,000 | **20.75** | **27.00** | **28.87** | **28.35** | **40.88** |  |
| 20,000-39,999 | **35.74** | **34.58** | **28.19** | **40.73** | **36.38** |  |
| 40,000-59,999 | **20.42** | **18.16** | **14.45** | **18.29** | **12.35** |  |
| 60,000-79,999 | **9.78** | **9.66** | **9.92** | **7.28** | **5.25** |  |
| 80,000+ | **13.31** | **10.60** | **18.57** | **5.35** | **5.14** |  |
| ***Dwelling ownership*** |  |  |  |  |  | ******* |
| Owned | **56.77** | **89.06** | **91.20** | **70.16** | **80.72** |  |
| Rent | **43.23** | **10.94** | **8.80** | **29.84** | **19.28** |  |
| ***Has mood disorder or not*** |  |  |  |  |  | ******* |
| No | **89.79** | **93.81** | **93.50** | **89.91** | **91.44** |  |
| Yes | **10.21** | **6.19** | **6.50** | **10.09** | **8.56** |  |
| ***Has anxiety disorder or not*** |  |  |  |  |  | ******* |
| No | **91.82** | **94.61** | **94.60** | **92.56** | **94.10** |  |
| Yes | **8.18** | **5.39** | **5.40** | **7.44** | **5.90** |  |
| ***Has chronic disease or not*** |  |  |  |  |  | ******* |
| No chronic disease | **26.21** | **29.09** | **33.47** | **19.62** | **23.70** |  |
| Has at least one chronic disease | **73.79** | **70.91** | **66.53** | **80.38** | **76.30** |  |
| ***Has difficulty in seeing or not*** |  |  |  |  |  | ******* |
| No | **80.30** | **85.16** | **82.88** | **72.43** | **79.41** |  |
| Yes | **19.70** | **14.84** | **17.12** | **27.57** | **20.59** |  |

**Appendix 1 continued.**

| ***Has difficulty in hearing or not*** |  |  |  |  |  | **n.s.** |
| --- | --- | --- | --- | --- | --- | --- |
| No | **77.93** | **76.85** | **80.24** | **75.16** | **76.21** |  |
| Yes | **22.07** | **23.15** | **19.76** | **24.84** | **23.79** |  |
| ***Has difficulty in walking or not*** |  |  |  |  |  | ******* |
| No | **63.15** | **74.67** | **75.75** | **57.14** | **63.28** |  |
| Yes | **36.85** | **25.33** | **24.25** | **42.86** | **36.72** |  |
| ***Has difficulty in selfcare or not*** |  |  |  |  |  | ******* |
| No | **93.26** | **96.02** | **94.45** | **87.62** | **89.32** |  |
| Yes | **6.74** | **3.98** | **5.55** | **12.38** | **10.68** |  |
| ***Has difficulty in remembering or not*** |  |  |  |  |  | ******* |
| No | **76.17** | **81.67** | **80.89** | **70.98** | **75.97** |  |
| Yes | **23.83** | **18.33** | **19.11** | **29.02** | **24.03** |  |
| ***Has difficulty in communicating or not*** |  |  |  |  |  | ******* |
| No | **95.61** | **95.98** | **95.00** | **90.95** | **92.49** |  |
| Yes | **4.39** | **4.02** | **5.00** | **9.05** | **7.51** |  |
| ***Has a usual place for immediate care***  ***for minor problem or not*** |  |  |  |  |  | ***** |
| No | **9.47** | **7.31** | **8.62** | **8.54** | **7.32** |  |
| Yes | **90.53** | **92.69** | **91.38** | **91.46** | **92.68** |  |
| ***Has a regular health care provider or not*** |  |  |  |  |  | ******* |
| No | **9.51** | **5.09** | **5.34** | **12.36** | **6.91** |  |
| Yes | **90.49** | **94.91** | **94.66** | **87.64** | **93.09** |  |

***Note*. n.s. is “not significant.” * p<0.05; ** p<0.01; *** p<0.001.**
